# Repair of complex ovine segmental mandibulectomy utilizing customized tissue engineered bony flaps

**DOI:** 10.1371/journal.pone.0280481

**Published:** 2023-02-24

**Authors:** Emma Watson, Hannah A. Pearce, Katie J. Hogan, Natasja W. M. van Dijk, Mollie M. Smoak, Sergio Barrios, Brandon T. Smith, Alexander M. Tatara, Timothy C. Woernley, Jonathan Shum, Craig B. Pearl, James C. Melville, Tang Ho, Issa A. Hanna, Nagi Demian, Jeroen J. J. P. van den Beucken, John A. Jansen, Mark E. Wong, Antonios G. Mikos

**Affiliations:** 1 Department of Bioengineering, Rice University, Houston, Texas, United States of America; 2 Medical Scientist Training Program, Baylor College of Medicine, Houston, Texas, United States of America; 3 Department of Biomaterials, Radboudumc, Nijmegen, The Netherlands; 4 Department of Oral and Maxillofacial Surgery, The University of Texas Health Science Center at Houston, Houston, Texas, United States of America; 5 Department of Otorhinolaryngology, The University of Texas Health Science Center at Houston, Houston, Texas, United States of America; University of Texas at San Antonio, UNITED STATES

## Abstract

Craniofacial defects require a treatment approach that provides both robust tissues to withstand the forces of mastication and high geometric fidelity that allows restoration of facial architecture. When the surrounding soft tissue is compromised either through lack of quantity (insufficient soft tissue to enclose a graft) or quality (insufficient vascularity or inducible cells), a vascularized construct is needed for reconstruction. Tissue engineering using customized 3D printed bioreactors enables the generation of mechanically robust, vascularized bony tissues of the desired geometry. While this approach has been shown to be effective when utilized for reconstruction of non-load bearing ovine angular defects and partial segmental defects, the two-stage approach to mandibular reconstruction requires testing in a large, load-bearing defect. In this study, 5 sheep underwent bioreactor implantation and the creation of a load-bearing mandibular defect. Two bioreactor geometries were tested: a larger complex bioreactor with a central groove, and a smaller rectangular bioreactor that were filled with a mix of xenograft and autograft (initial bone volume/total volume BV/TV of 31.8 ± 1.6%). At transfer, the tissues generated within large and small bioreactors were composed of a mix of lamellar and woven bone and had BV/TV of 55.3 ± 2.6% and 59.2 ± 6.3%, respectively. After transfer of the large bioreactors to the mandibular defect, the bioreactor tissues continued to remodel, reaching a final BV/TV of 64.5 ± 6.2%. Despite recalcitrant infections, viable osteoblasts were seen within the transferred tissues to the mandibular site at the end of the study, suggesting that a vascularized customized bony flap is a potentially effective reconstructive strategy when combined with an optimal stabilization strategy and local antibiotic delivery prior to development of a deep-seated infection.

## Introduction

Repair of large craniofacial defects can be challenging due to the need for a strategy that yields functional and aesthetic results. The current reconstructive gold standards of a vascularized osteocutaneous flap or non-vascularized particulated autogenous bone can result in donor site morbidity [1], and the commonly used vascularized flaps such as a fibular or scapular free tissue transfer are often a poor approximation for the craniofacial bone being replaced [2]. To overcome these issues, a two-stage approach to craniofacial reconstruction is proposed [3]. In the first stage, a space maintainer is implanted into a defect site to prevent the infiltration of fibrovascular tissue and to optimize the soft tissue pocket for later reconstruction; simultaneously, a 3D printed customized bioreactor is implanted adjacent to a periosteal surface at a distant site. After bony tissue has formed in the bioreactor, a second surgery is conducted during which the space maintainer is removed, and the newly generated bioreactor bone is transferred as a flap with vasculature or as an avascular graft.

To determine the efficacy and feasibility of such a strategy, a series of ovine models have been developed. The earliest models merely evaluated for bone growth within the bioreactors [4]; however, more recent studies have involved the transfer of bioreactor-generated tissue to various mandibular sites. The first site selected for transfer was the angle of the mandible [5, 6]—a location close to the large neck vessels facilitating anastomoses and relatively protected within the neck from the bacteria-filled oral cavity or extensive forces of mastication. After demonstrating that the generated tissue could be transferred as a vascularized flap and that integration occurred with the native mandibular bone, the complete two-stage strategy was successfully performed in a sheep [3]. Next a superior partial segmental defect was created in the edentulous region of the ovine mandible and filled with a porous non-antibiotic-loaded space maintainer while bioreactors were implanted adjacent to rib periosteum. After 9 weeks, the bioreactor tissues were transferred as vascularized flaps to the partial segmental defect. This model was more challenging in that it was immediately adjacent to the thin oral mucosa, further from the large vessels of the neck, and likely experienced significant mechanical forces during mastication. As facial trauma and the removal of benign or malignant mandibular tumors often require excision of a complete portion of the mandible [7], a third, more challenging, defect was developed, in which a total segmental defect was created. This produces complete segmental defects that are exposed to significant mechanical forces and are immediately adjacent to the oral cavity.

In addition to increasing the complexity of our defect site, the geometry and fabrication methods of the *in vivo* bioreactors have also been improved to allow for the generation of customized bony segments. Early studies involved the use of dental wax around which poly(methyl-methacrylate) (PMMA) was molded, and boiled clean after curing [8]. This allowed for the production of bioreactors that could generate long rectangular tissues of 4 x 1 x 1 cm^3^. This molding technique was also utilized to create bioreactors that mimicked the size and shape of the mental protuberance [9]. The advent of 3D printing can allow for the creation of more complex bioreactors from computed tomography scans of patients with increased resolution over molding. While early studies utilizing 3D printing still utilized simple rectangular bioreactors [3, 10], this study utilized a more complex bioreactor with a central protuberance to allow for creation of a channel within the bony tissue through which a neurovascular bundle could run.

To evaluate the two-stage strategy in a large, load-bearing, clinically relevant defect, we here utilized a complete segmental ovine defect model in this study. The defect was the same length as the previously evaluated superior hemisegmental defect (~2 cm [3, 10]) but involved the removal of a full segment. The remaining mandibular portions were stabilized similarly to a human segmental mandibular defect, i.e. with a single lateral titanium plate (of ~2.5 mm thickness) and 7–11 mm long bicortical screws [11]. We utilized two different bioreactor geometries, a small bioreactor (2 cm x 0.75 cm x 1 cm) of a simple rectangular geometry and a large bioreactor (2 cm x 1.5 cm x 1 cm) with central protuberance to generate the tissue needed for repair. We hypothesized that (i) the single lateral plate would be sufficient for mandibular stabilization and space maintainer retention, (ii) the bioreactors would all generate bony tissue allowing transfer as a vascularized flap, and (iii) that the procedure would result in successful repair of the mandibular defect, yielding similar radiographic and histologic scoring results compared to a contralateral unoperated control.

## Materials and methods

### Bioreactor and space maintainer fabrication

Bioreactors were created from poly(methyl-methacrylate) using an extrusion 3D printer as previously described [3, 10]. Two different bioreactor geometries were investigated. The smaller bioreactors were 2 cm x 0.75 cm x 1 cm and completely hollow, while the larger bioreactors were 2 cm x 1.5 cm x 1 cm and had a central protrusion (2 mm x 3 mm) that would allow for the inclusion of a groove in the formed tissue to accommodate the inferior alveolar nerve passing through the mandible in the segment removed. An ethylene-vinyl acetate cuff was heat-molded around each bioreactor prior to sterilization with ethylene oxide. Images and further description of the bioreactors is given in S1 Fig.

Space maintainers were fabricated as previously described [12, 13]. A carboxymethyl cellulose (CMC, Spectrum Chemical, New Brunswick, NJ) gel was formed at 9 wt% in water. Space maintainers were created with 30 wt% CMC gel and bone cement powder and bone cement liquid at 2:1 w/v ratio. The powder phase (84 wt% polymerized methyl-methacrylate/methyl-acrylate copolymer, 1 wt% benzoyl peroxide, and 15% zirconium dioxide, kindly donated by Synthasome, Inc., San Diego, CA) and CMC were mixed together to form a homogeneous paste. The liquid phase (97.5 wt% methacrylate monomer, 2.5 wt% N,N-dimethyl-4-toluidine, and 75 ppm hydroquinone, Synthasome, Inc.) was then added and stirred. The dough was transferred to 3D-printed molds (Dental SG resin, FormLabs) of 2 cm x 1.5 cm x 1 cm and allowed to cure. After 24 hours of curing, the scaffolds were leached for 3 days in excess water. Space maintainers were vacuum-dried and sterilized with ethylene oxide.

### Mandibular defect creation and bioreactor implantation

The animal protocol was approved by the Animal Welfare Committee of the University of Texas Health Science Center (AWC 19–0015), the Institutional Animal Care and Use Committee at Rice University (1463571), and the Animal Care and Use Review Office of the US Department of Defense (AF130059-CF). Five female Dorper sheep (K Bar Livestock, LLC, Sabinal, TX) aged 6–9 months and weighing 29.5 ± 1.2 kg at the time of the first surgery were anesthetized using intravenous telazol and inhaled isoflurane. Bioreactor implantation occurred as previously described [3, 5, 6, 10, 14]. Briefly, two incisions were created over ribs 4 and 8, and subsequently ribs 3, 5, 7, and 9 were exposed by blunt dissection. A 3 cm bony segment from each of these ribs was removed and morselized to create autograft. Bioreactors were filled with 50:50 v/v autograft and xenograft (Bio-Oss^®^, kindly donated by Geistlich), prior to implantation adjacent to the periosteum. A larger bioreactor was always implanted adjacent to rib 5 periosteum (the bioreactor tissue intended for transfer), and the locations of the other 3 bioreactors (2 small and 1 large) were randomized.

**Fig 1:**
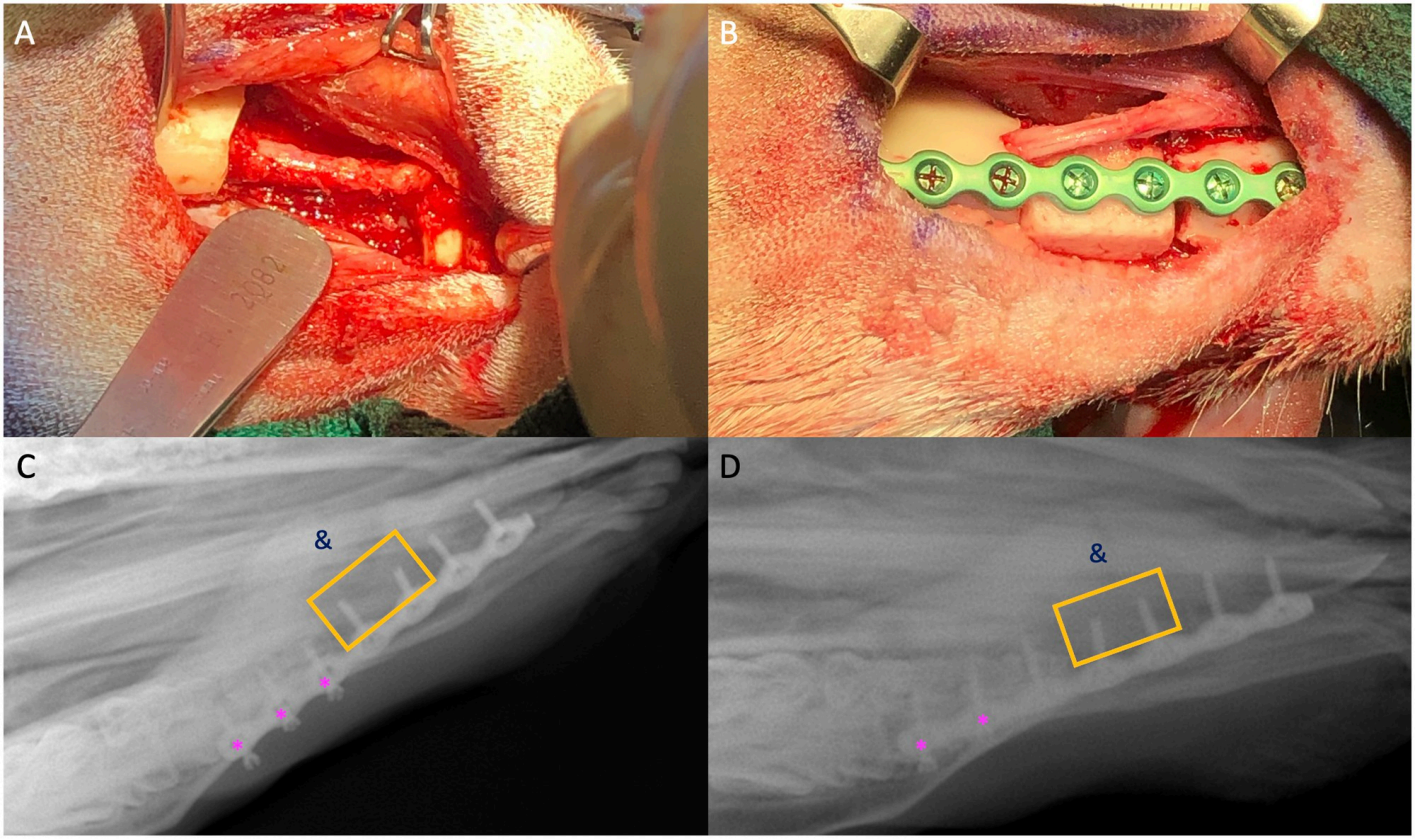
Successful space maintainer implantations with single lateral plate. The creation of the defect showing preserved mental nerve (A); the space maintainer was shaped to the defect, and a groove was placed anteriorly for the nerve to run across (B); and X-rays showing position of space maintainer (yellow box), medial callus formation (&), and loosening screws (pink asterisks) for two different sheep (C,D).

The mandibular defect was created through a 6 cm incision at the lower border of the mandible in the edentulous region of the diastema. After blunt dissection to expose the mental nerve anteriorly and the most anterior molar posteriorly, a window was created into the inferior alveolar nerve canal. Then, a bicortical (buccal and lingual plates) defect was created superior to the nerve canal, prior to plating the mandible with a laterally placed reconstruction plate (KLS Martin, Tuttlingen, Germany), which was secured with a minimum of 2 bicortical screws anteriorly and 3 posteriorly (KLS Martin). Following attachment of the plate, the remaining inferior border of the mandible was removed, creating a full-thickness, complete segmental defect while preserving the mental nerve (Fig 1A and S2 Fig). The space maintainer was trimmed to fit the defect site with a dental bur under irrigation, and the edges were rounded to prevent sharp edges adjacent to the overlying oral mucosa. The space maintainer was then secured in place with two screws (Fig 1B), and the incision was closed. One animal received two plates, a lateral plate and an inferior plate (KLS Martin) after an earlier sheep experienced hardware failure with the single lateral plate. The plate arrangements and screw lengths for each sheep are given in S1 Table.

### Animal monitoring and treatment

Sheep were monitored daily by veterinary staff throughout the course of the 21-week study for intakes and outputs, and the incisions were checked regularly for signs of infection. Weights were taken prior to both surgeries. Concerns about animal status were reported to the large animal veterinarian, who then discussed concerns and potential modifications with the surgical team, comprised of clinicians who had all participated in a sheep-specific course offered by the large animal veterinarian at the University of Texas Health Science Center.

Animals with signs of infection had swabs taken, and culture results were used for appropriate antibiotic selection; either Excede (ceftiofur), penicillin G, Baytril (enrofloxacin), or metronidazole, or a combination were utilized. Buprenorphine was administered to animals showing excessive bleating, decreased appetite, or other signs of pain, at the discretion of the veterinarian. If there were concerns for hardware failure and infection, sheep were sedated with telazol, and radiographic images of the mandible were taken to view the hardware. Sheep were switched to a pellet-based diet to lessen need for chewing and rumination after several sheep experienced dehiscence. In addition to the aforementioned modifications and treatments, humane endpoints were utilized to remove two animals from the study (with 3 animals successfully completing the study and no animals dying prior to removal from the study).

Humane endpoints were based on behavioral changes (decrease in appetite, increase in vocalization, altered social interaction), clinical appearance (volume, color, and frequency of discharge), and available treatment options (spectrum of antibiotics tried, ability to restore stability to the mandible segments). For the first sheep displaying increased vocalization with purulent discharge and was determined to have a fractured fixation plate, surgery was attempted to replace the plate; however, it was noted that the mandible cortex in the areas of fixation was comminuted and required more than a simple plate replacement. Under intraoperative guidance with the veterinarian, this animal was humanely euthanized. The second animal that showed increased vocalization and continued purulent discharge despite treatment with antibiotics providing gram-positive, gram-negative, and anerobic coverage, was also removed from the study a week after surgical debridement after discussion with the veterinarian as the discharge continued to worsen.

### Mandibular reconstruction and bioreactor harvest

After 9 weeks, the reconstruction surgery was performed as previously described [3]. The bioreactor on the fifth rib was utilized for the reconstruction, assuming bone growth could be observed and the vascular supply could be located. Briefly, two incisions were created over ribs 4 and 8, the bioreactors on ribs 3, 5, 7, and 9 were located by blunt dissection. The bioreactor on rib 5 was isolated with its adjacent intercostal artery and vein. All other bioreactors were harvested without vasculature and fixed in 10% neutral buffered formalin prior to analysis of bone formation characteristics.

An incision was created at the inferior border of the mandible. The lateral plate, bicortical screws, and space maintainer were removed. In the event of an infection around the space maintainer, the defect area was debrided and irrigated with copious amounts of normal saline. If a mucosal dehiscence was present, the mucosal opening was sutured closed. The vascular anastomoses were performed to branches of the facial artery and vein using a surgical microscope (Leica Microsystems). As all sheep had developed a robust callus medially that provided sufficient mechanical support for the mandible, smaller monocortical self-tapping screws and lateral plate (as in the partial segmental defect [3, 10]) were utilized to hold the transferred bioreactor tissue in place.

### Mandible harvest

The sheep were euthanized 12 weeks after mandibular reconstruction. The entire mandible was harvested as previously described [14]. After administration of pentobarbital/phenytoin, the mandible was removed. A diamond blade saw was utilized to isolate the reconstructed segmental defect in the region of the edentulous area of the mandible. Tissues were fixed in 10% neutral buffered formalin for 1 week prior to long-term storage in 70% ethanol.

### Microcomputed tomography

Bioreactor and mandibular specimens were imaged with a SkyScan 1272 microCT imaging system (Bruker, Billerica, MA) as previously described [10]. The contralateral unoperated mandible served as the mandibular control, and non-implanted bioreactors packed with the 50:50 v/v autograft:xenograft mixture were utilized as the time zero bioreactors. Scans were performed with a rotation step size of 0.4°, frame averaging of 4, random movement of 10, and pixel size of 16.2 μm with 360° scanning. NRecon (version 1.7.4.6; SkyScan) was used for the reconstruction and performed with a threshold of 0.002–0.032 (dimensionless). For the bioreactors, the excised region that underwent reconstruction, and the contralateral mandible, CTAn (version 1.18.8.0) was used to analyze the bone volume to total volume ratios (BV/TV), the trabecular thickness (TbTh), the trabecular number (TbN), and the trabecular spacing (TbSp).

The ratio of autologous bone (defined as implanted autograft and newly formed bone) to xenograft was measured using ImageJ (version 1.52p, NIH) as previously described [10]. Briefly, the reconstructed bitmap files were analyzed with the MorphoLibJ plugin for extended minima and maxima with connectivity of 4 and threshold of 108 to select only for xenograft. The area of the pixels was measured. Slices at 0.5, 1, and 1.5 cm along the 2 cm bioreactor were used for analysis.

### Mechanical testing

Bioreactor specimens were bisected with a diamond blade saw, and halves were randomly assigned to undergo either mechanical testing or histological analysis. The compressive mechanical testing was performed as previously described [3, 10]. Briefly, a diamond-blade saw was utilized to ensure that no aspect ratio exceeded 2:1. An Instron 5565 and 2kN load cell with a crosshead speed of 1 mm/min was used, and the modulus and offset yield were calculated on data generated within Instron Bluehill 3 testing software.

### Histological processing and analysis

Fixed mandibular specimens and remaining bioreactor halves were analyzed as previously described [3]. Specimens were methacrylate embedded after serial dehydration prior to sectioning with a diamond blade (Leica Microsystems SP 1600). Three 10–15 μm sections from each specimen were stained with methylene blue and basic fuchsin. Mandibular sections were made through the posterior screw within the transferred bioreactor tissue, while bioreactor sections were taken at the midpoint of the total length of the specimen.

A panel of blinded scorers utilized established scoring matrices (S2 and S3 Tables) to score stained sections of each mandibular or bioreactor specimen [3, 6, 14]. Mandible sections were scored for scaffold coverage percentage, hardware osteointegration, bony bridging, mucosal intactness, and presence of inflammatory cells and a periosteal reaction. Bioreactor specimens were analyzed for scaffold coverage and bone maturity, along with the presence or absence of osteoclasts. The median score was used for statistical analysis [15]. ImageJ was utilized to determine the fractional depth of the bioreactor tissues measuring the distance between the periosteum and furthest viable osteocyte at 1/3, 1/2, and 2/3 of the length of the periosteum and calculating the mean.

### Statistics

Physical, biologic, radiographic, and histomorphometric statics were computed using a one-way ANOVA with post hoc Tukey’s honestly significant difference (α = 0.05). Histological scores were examined with a Kruskal-Wallis test with post hoc analysis via the Steel-Dwass test. Assay results from animals that were euthanized prior to the intended time points are presented in S3 Fig and are not included in the statistical analysis to determine significance. Statistics were performed using JMP Pro-15.0 (SAS, Cary, NC).

## Results

### Mandibular defect creation and bioreactor implantation outcomes

All sheep tolerated the surgical procedures for mandibular defect creation and bioreactor implantation well and resumed hay or pellet diets the day of the surgery. The surgeries for the mandibular defect creation and space maintainer implantation required modifications between sheep (S1 Table). In the first sheep, a 3.0 mm thick titanium fixation plate was utilized; however, the plate was very large relative to the size of the defect and incision, so a thinner 2.5 mm plate was utilized for future sheep (S1 Table and Fig 1B).

**Fig 2:**
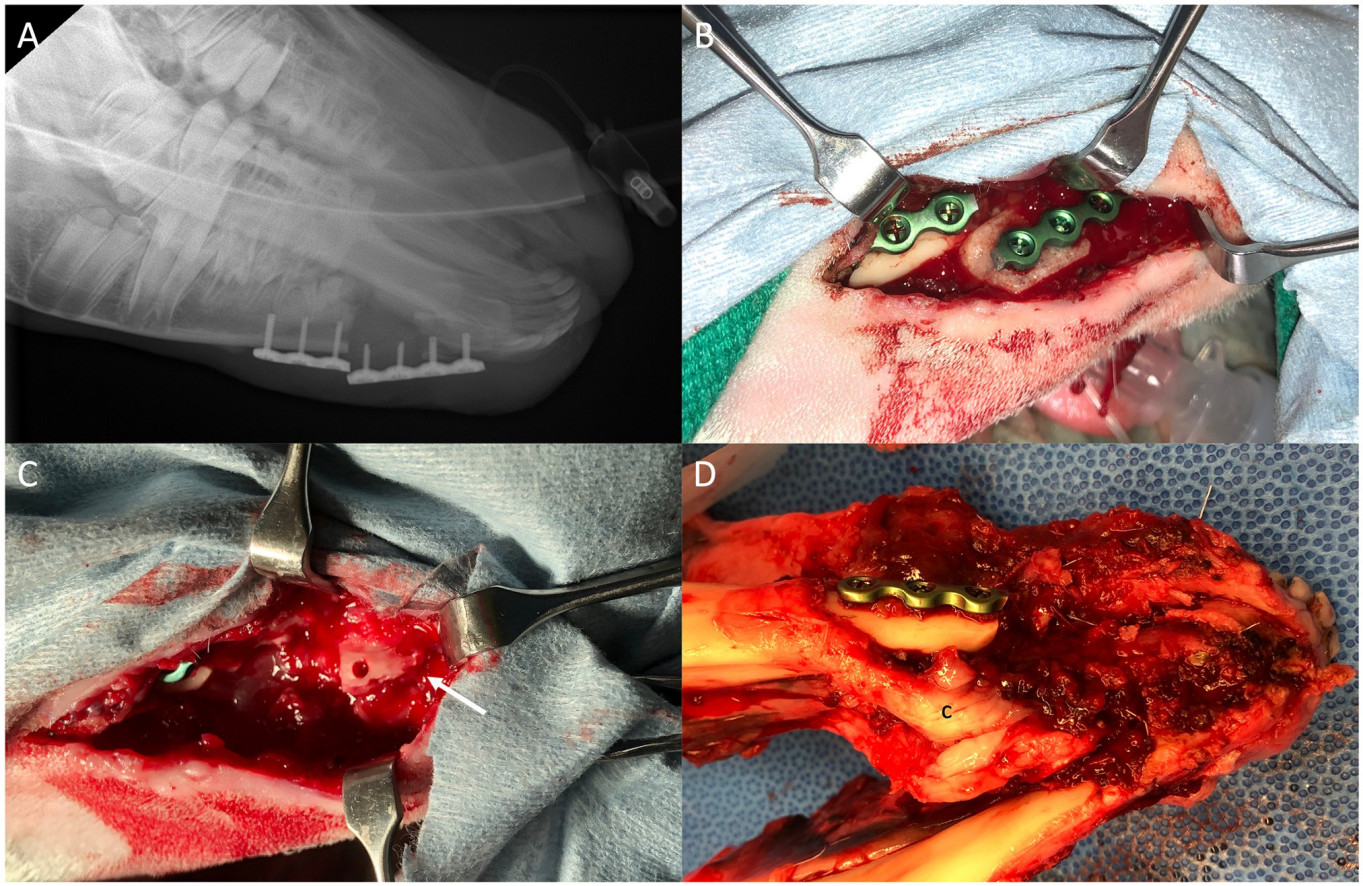
The removal of sheep from the study due to plate fracture. Pre-operative x-rays taken prior to attempted repair of unstable mandible (A); the plate fracture as visualized in the operating room (B); the fractured anterior mandible indicated by arrow (C); and the mandible removed after necropsy showing medial callus formation (c) and badly fractured anterior mandible (D).

Two sheep showed signs of infection at the incision site two weeks after the implantation surgery. The swab of one sheep (Sheep 1—the sheep with the large 3.0 mm fixation plate) cultured gram-positive cocci in clusters, but the mandible was stable. The other sheep had palpable crepitus of the mandible, and a revision surgery was conducted 4 weeks after the implantation surgery. Pre-operative X-rays taken of the sedated sheep showed a plate fracture (Fig 2A). During the surgery, it was discovered that the distal segment (anterior mandible) had been too badly fractured for re-plating (Fig 2B and 2C), and the animal was euthanized.

**Fig 3:**
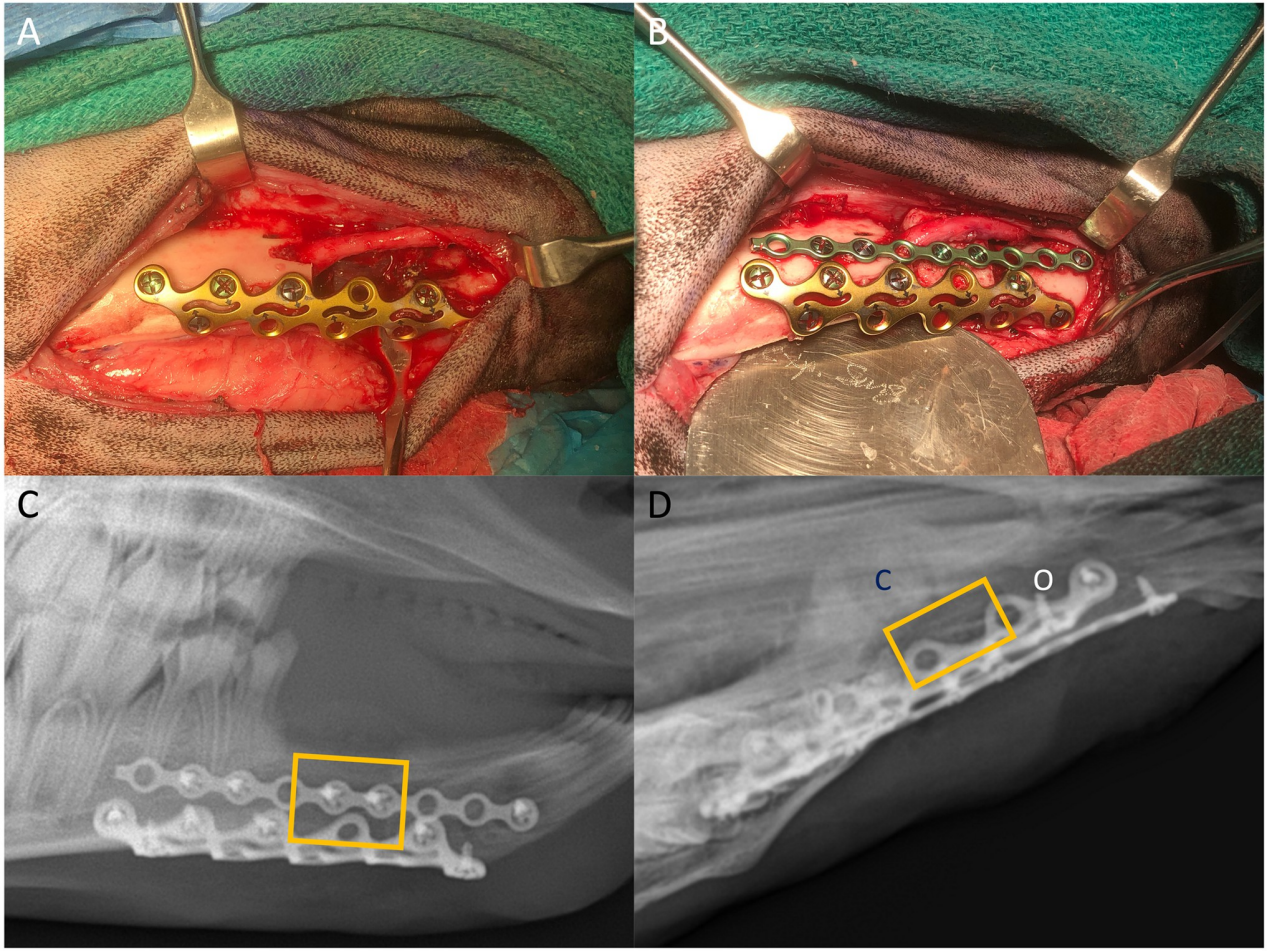
The two-plate strategy to provide additional inferior support to the mandible. Plating of the mandible with the rib plate (A), and a small lateral plate was used to hold the space maintainer in place (B). Initial X-rays (C) showed two plates and space maintainer (yellow box). Week 7 x-ray (D) showing medial callus formation and potential osteolysis associated with mucosal dehiscence observed at week 5.

At this point, following guidance from the veterinarian, it was decided that all sheep were required X-rays to check the status of the hardware. Screw loosening and callus formation were observed in the other three sheep (Fig 1C and 1D), and the sheep were switched to a pellet diet to limit chewing. Given the catastrophic hardware failure in one sheep and the less-severe hardware failure in the three-remaining sheep, the plating strategy was altered for the replacement sheep (Fig 3 and S1 Table). The two-plate strategy was completed successfully. Serial X-rays still showed medial callus formation but no significant loosening of screws (Fig 3C). However, this animal also showed signs of infection around 5 weeks post-implantation, and later X-rays showed areas of osteolysis and decreased radio-opacity (Fig 3D) around the anterior edge of the inferior plate. Four sheep underwent the bioreactor transfer and reconstruction.

### Mandibular reconstruction outcomes

**Fig 4:**
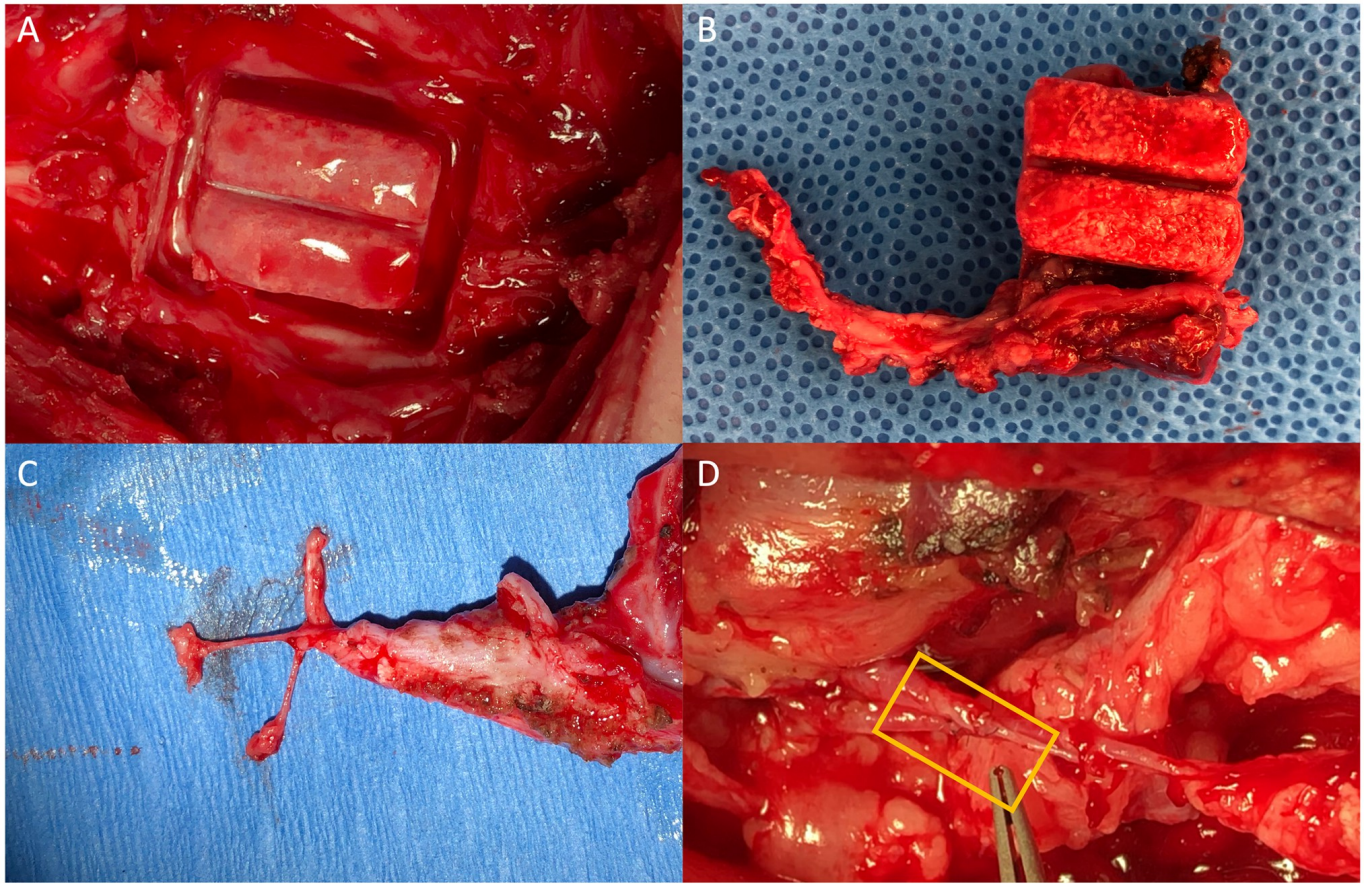
Bioreactor harvest and mandibular reconstruction. Appearance of the bioreactor site after removing the bioreactor from the newly generated bony tissue (A) the grooved tissue after removal along with the vasculature (B), the isolation of the nerve, artery, and vein located within the pedicle (C), and the microvasculature anastomosis (D) of the flap (left) with the systemic circulation (right) with yellow box showing sutured anastomosis.

**Fig 5:**
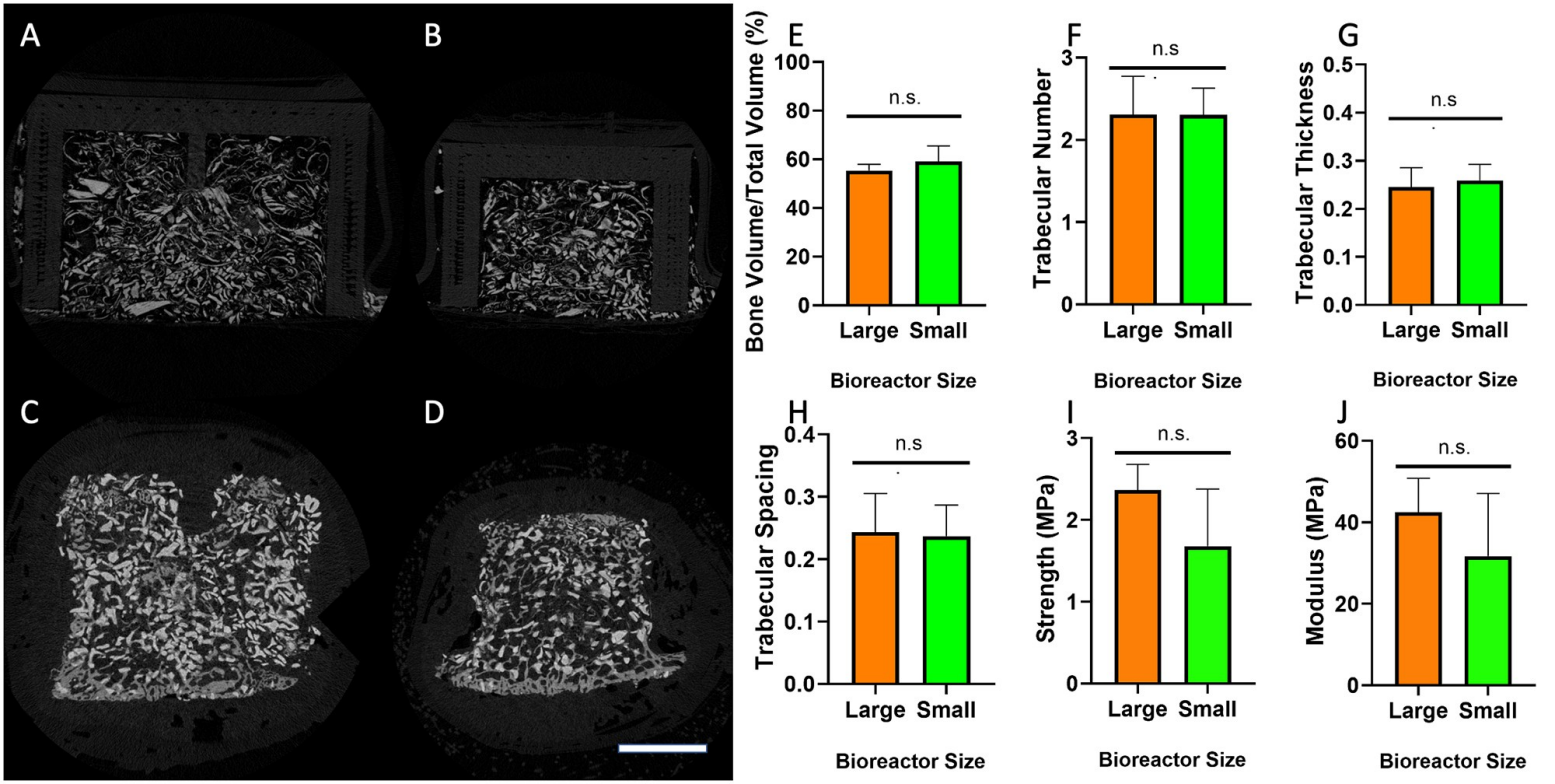
Bioreactor tissue analysis. Representative microCT images of bioreactor tissues from large (A,C) and small (B,D) bioreactors prior to implantation (A,B) and after 9 weeks implantation (C,D); Scale bar: 500 μm. Radiographic analysis of tissue in the large and small bioreactors after 9 weeks of implantation showing BV/TV (E), TbN (F), TbTh (G), and TbSp (H). Mechanical analysis of specimens allowed for the calculation of compressive modulus (I) and compressive strength (J) were calculated for bioreactor tissue harvested at 9 weeks. (n.s. = not significant, p > 0.05).

The mandibular reconstruction surgery was successfully conducted in four sheep. At the time of the surgery, one sheep had an external skin dehiscence, two sheep had mucosal dehiscence, and one sheep had no dehiscence. The bioreactors on the fifth rib all grew bony tissue (Fig 4A), and this bioreactor was utilized for the transfer as a vascularized flap in 3 of the 4 animals (Fig 5B and 5C). In one animal, the vascular supply of the rib 5 bioreactor could not be located, so the large bioreactor on rib 7 was used instead for transfer. In the animals with infection, surgical debridement was conducted and copious amounts of irrigation were used in an effort to remove the infected tissue, and new hardware was used to hold the vascularized tissue in place. The microvascular anastomoses were successfully performed in all animals, and patency verified with arterial pulsations (Fig 4D). All 16 bioreactors removed at 9 weeks contained tissue; however, two bioreactors broke apart due to lack of bone ingrowth during harvest, so 14/16 bioreactors grew tissue mechanically robust enough to transfer. All animals returned to pellet diet the day of surgery. One sheep (the sheep initially plated using the 3.0 plate) still showed excessive purulent discharge from an external dehiscence 1-week post-transfer, even after extensive surgical debridement and antibiotics with gram-positive, gram-negative, and anaerobic coverage. Therefore, the veterinary consultation recommended removal from the study. Of the 3 animals remaining in the study, 1 animal showed no signs of infection, and the other two animals were intermittently treated for infection by the veterinarian. One animal developed an additional external dehiscence after the reconstruction surgery; however, these infections were not severe enough to warrant veterinary removal from the study.

### Bioreactor radiographic and mechanical analysis

All bioreactor tissues underwent radiographic analysis, including those that were harvested from the sheep euthanized at 4 weeks (Fig 5 and S4 Table). There were no significant differences between BV/TV, TbN, TbTh, or TbSp for the large or small bioreactors (p > 0.05). The BV/TV for the large and small bioreactors was 55.3 ± 2.6% and 59.2 ± 6.3%, respectively. Cross-sections from samples prior to implantation showed bright granules surrounded by less-radio-dense thin strips of morselized bone. After 9 weeks of implantation, additional new bone formation could be observed surrounding the bright granules. Compressive mechanical analysis was performed on both the samples harvested at 9 weeks as intended and those removed from the sheep that was euthanized prematurely secondary to plate fracture (Fig 5 and S4 Table). The compressive modulus and strength showed no significance difference between the large and small bioreactors (p > 0.05). The 4-week bioreactors had compressive moduli below 10 MPa and strengths below 1 MPa, while the 9-week tissues showed compressive moduli of 31.7 ± 15.4 MPa (small) and 42.5 ± 8.3 (large) and strengths of 1.68 ± 0.70 MPa (small) and 1.63 ± 0.31 MPa (large).

### Mandibular radiographic analysis

**Fig 6:**
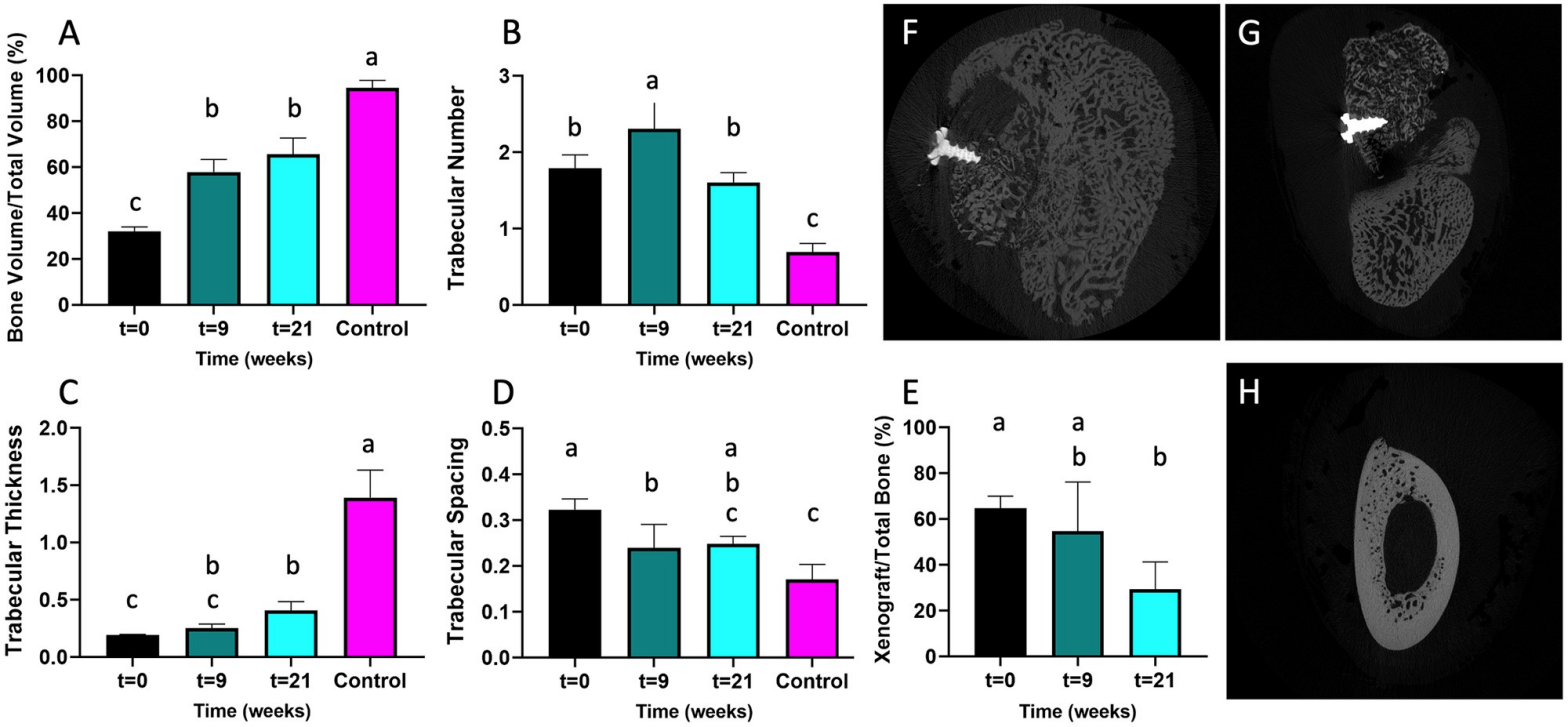
Remodeling of tissues with time. MicroCT was utilized to measure the BV/TV (A), TbN (B), TbTh (C), and TbSp (D) over time, with the t = 0 representing bioreactors pre-implantation (n = 6), t = 9 the bioreactors at the transfer surgery (n = 12), t = 21 the mandibles of the sheep who successfully completed the study (n = 3), and the control representing the contralateral unoperated mandible (n = 4). Image analysis was utilized to determine the proportion of xenograft with time (E). Representative radiographic cross-sections show modest osseointegration of the transferred bioreactor tissue (F) or good hardware integration but poor integration with adjacent bone (G), and the contralateral control mandible (H). Those that do not share the same letter are significantly different (p < 0.05).

Radiographic analysis was also performed on the reconstructed mandibular site of the area of the transferred bioreactor tissue and on the contralateral control mandible at the same location (Fig 6), including determination of BV/TV, TbN, TbTh, and TbSp. At t = 21 weeks, the intended endpoint of the study, the mandibular bioreactor tissue BV/TV was 64.5 ± 6.2%. This was not significantly higher than all the bioreactors at t = 9 weeks (57.3 ± 5.1%, p > 0.05), but it was significantly higher than the bioreactors pre-implantation (31.8 ± 1.6%, p < 0.05). Although the BV/TV has increased since implantation, the BV/TV was still significantly lower (p < 0.05) than that of the contralateral control (94. ± 3.4%) (Fig 6A). The trabecular spacing decreased with time, while the trabecular thickness increased with time, indicating continued remodeling and maturation. The ratio of xenograft to total bone (Fig 6E) was also calculated. Before implantation, the xenograft to total bone ratio was 64.7 ± 5.3% as measured by microCT; after 21 weeks of implantation, it was significantly lower 22.0 ± 17.6% (p < 0.05). Although the sheep without signs of infection showed higher degree of osseointegration of the screw and of the transferred bioreactor tissue with the adjacent callus (Fig 6F), animals with signs of infection displayed poor integration with the adjacent bone (Fig 6G). The geometry of contralateral control mandible (Fig 6H) was not recapitulated in the reconstructed mandibles after 21 weeks, with the latter showing and decreased bone density.

### Histological analysis

**Fig 7:**
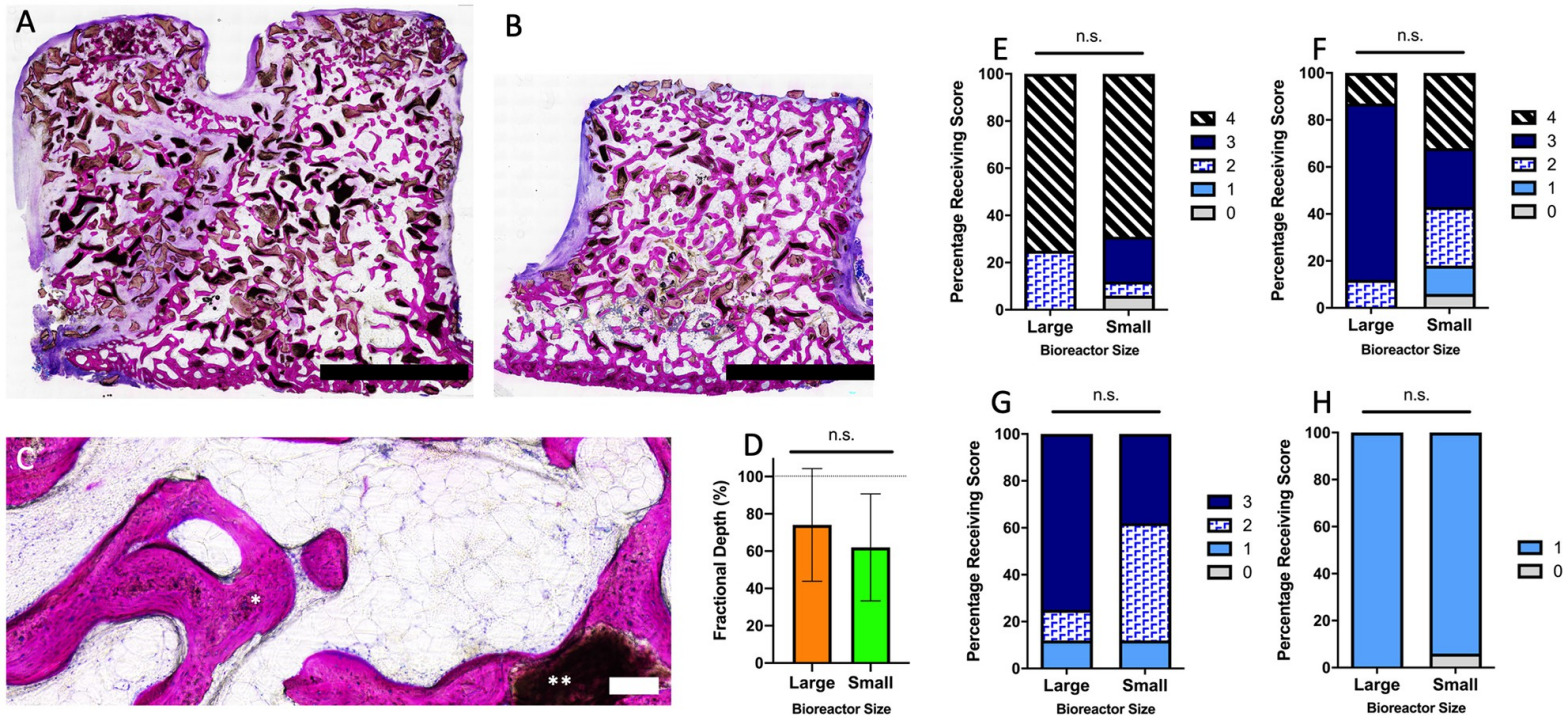
Histological analysis of bioreactor tissues. Low magnification images are shown of tissue in the large (A) and small (B) bioreactors. A high magnification image (C) shows bone forming around autograft and xenograft within the bioreactors. Using these images, the fractional depth (D) was measured, and a blinded panel of histological reviewers were used to determine the autograft coverage (E), xenograft coverage (F), tissue maturity (G), and osteoclast presence (H). Scoring was based on n = 4 large bioreactors and n = 8 small bioreactors. Scale bars: A & B = 5 mm, C = 100 μm. * = remodeling autograft; ** = xenograft in C. n.s. = non-significant (p > 0.05).

The bioreactors harvested at 9 weeks that were not used for mandibular reconstruction were processed for histological analysis. There was no significant difference (p > 0.05) in the relative area of autograft coverage, area of xenograft coverage, tissue maturity or presence of osteoclasts between the large and small bioreactors (Fig 7). There were also no differences in the fractional depth, with the bony tissue in the large bioreactors reaching a fractional depth of 74.1 ± 30.2% and in the small bioreactors 62.0 ± 28.7% (Fig 7). Comparing these bioreactors to those harvested from the sheep prematurely euthanized at 4 weeks (S3 Fig), there were significant increases in autograft coverage (p = 0.01) and tissue maturity (p = 0.02), but no significant difference in xenograft coverage (p = 0.07). The fractional depth for both bioreactor sizes also increased significantly from 4 weeks to 9 weeks implantation, from 21.0 ± 4.2% to 67.0 ± 29.0% (p = 0.002).

**Fig 8:**
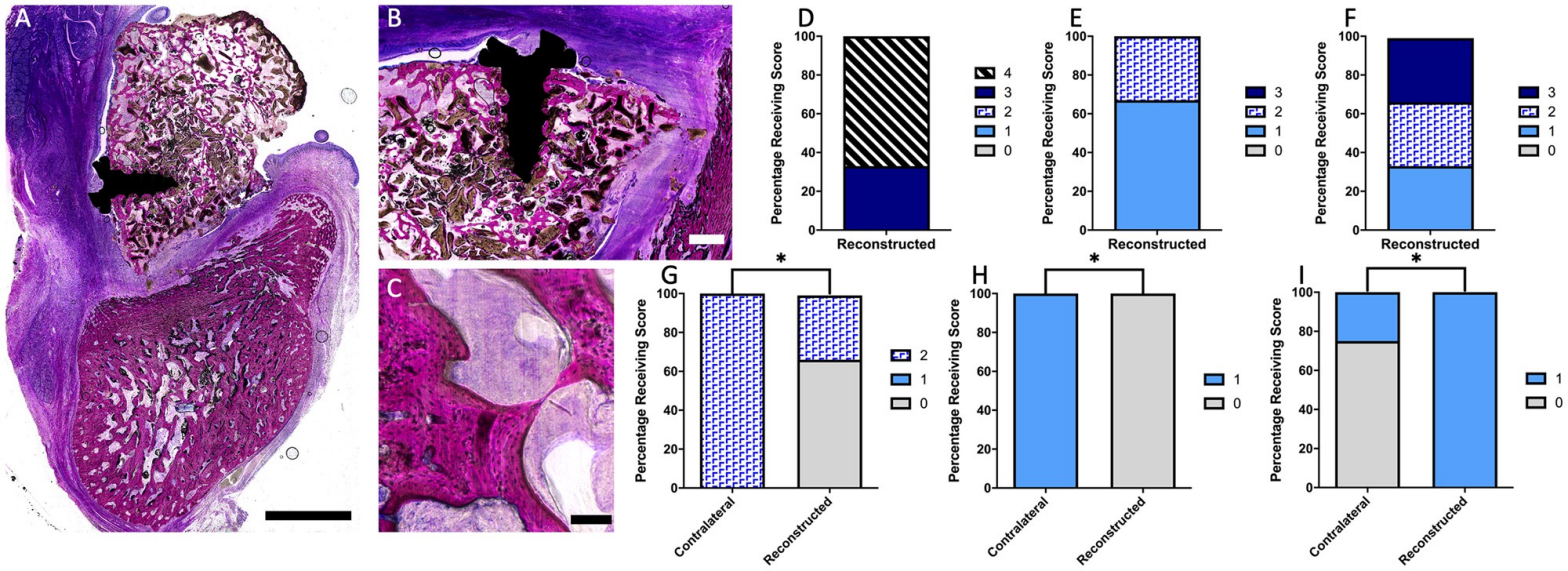
Histological analysis of mandibles. Low magnification (A) image of reconstructed mandible, with high magnification images showing clear signs of robust osseointegration of the screw (B) and viability of bony tissue (C). The graft coverage (D), integration of bony tissue with the adjacent native mandible (E), and screw osseointegration (F) are given for the n = 3 reconstructed mandibles. The mucosal intactness score (G), inflammatory cell presence (H), and periosteal reaction (I) scores are given for both the reconstructed (n = 3) and control (n = 4) mandibles. Scale bars: A = 5 mm, B = 1 mm, and C = 100 μm. * indicates significant difference between the two groups (p < 0.05).

The histological analysis of reconstructed mandibula (Fig 8, control mandible histology given in S4 Fig) showed a significant decrease in mucosal intactness score and increase in the presence of inflammatory cells when comparing the reconstructed to contralateral control sites (p < 0.05). Three animals showed at least partial integration of the screw within the transferred flap, and one animal did not. The graft coverage was > 75% in 3 out of 4 of the animals. The mandibular geometry of the reconstructed site was grossly enlarged, and this increase in size required removal of bony tissue away from the flap for sectioning and mounting, preventing the measurements of area, height, and width of the mandible as reported previously [10].

## Discussion

The aim of this study was to challenge the two-stage repair strategy, combining space maintainer technology at the mandibular defect site, generating vascularized bone in a distant bioreactor, and transfer of the latter to the mandibular defect in a large, load-bearing, clinically-relevant ovine model. We hypothesized that (i) the single lateral plate would be sufficient for mandibular stabilization and space maintainer retention, (ii) the bioreactors would all generate bony tissue allowing transfer as a vascularized flap, and (iii) that the procedure would result in successful repair of the mandibular defect, yielding similar radiographic and histologic scoring results compared to a contralateral unoperated control. Although the plating strategy was sufficient to maintain the space maintainer within the defect site, hardware failure and dehiscence created a challenging environment for reconstruction. Both large and small bioreactors demonstrated substantial vascularized bone formation at 9 weeks. After transfer, the newly formed bone continued to remodel; however, the transferred tissue never attained the radiographic properties of the contralateral control mandible, likely due in part to the infection present in many of the defect sites.

Although the goal of a scientific study should be to eliminate variability between test groups or animals, sometimes small adaptations must be made, especially when animals reach humane endpoints before the study endpoint. Initially, the plating strategy adopted in this animal model was a single reconstructive plate on the lateral surface of the mandible with bicortical screws to provide additional support and reduce the cortical bone stress [16]. Sheep are ruminants and constantly chew and rechew their food to reduce the particle size for digestion [17]. While ruminating, sheep produce large lateral movements [18, 19], and other studies have noted significant differences between the chewing patterns of humans and sheep [20, 21]. The plate fracture occurred in the animal that received two “emergency screws” (larger diameter of 2.3 mm), which are used when the original screw holes are stripped and are unable to retain the intended screws clinically and provide superior screw fixation [22]. While a single lateral plate has been successfully utilized in a similar ovine defect [23], other researchers have used a customized porous titanium plate that offers support to multiple locations along the mandible [24]. This approach, however, eliminates the contact between the porous space maintainer and the overlying oral mucosa that we were also hoping to study. To counter this, a 3-dimensional plate that provided stability along the inferior lingual and buccal cortices with a thinner lateral plate to hold the space maintainer in place was utilized. No hardware failure was observed on subsequent imaging. While these animal-to-animal changes could be looked upon as a limitation of the study, they allowed for the development of an adequate fixation strategy and revealed an important consideration when attempting stabilization of segmental defects in ruminants—fixation should extend along the lateral, medial, and inferior surfaces of the jaw bone.

All sheep developed a large callus medial to the defect site that spanned the entire length of the defect and provided enough stability to the mandible that the large plate initially utilized for fixation was no longer required after the reconstructive surgery. Micromotion leads to increased callus formation [25], and due to screw failure in 3 of 4 animals that underwent the second surgery, it is evident that the fixation was not adequate. In the animal that received the two plates, no outright fixation failure was observed, but a medial callus still formed. Previous studies have demonstrated a significant increase in mandibular area without infection after the creation of the partial segmental defect with a periosteal reaction noted on histology [3]. It is possible that the intact periosteum contributed to the large callus formation in these animals.

Infection was a common complication observed in this study, seen in 4 of 5 animals at the reconstructed mandibular site and associated with both mucosal and external dehiscence. Attempts to treat the hardware- and implant-associated infections with systemic antibiotics proved largely unsuccessful, with treatments decreasing or thinning exudate and reducing swelling rather than completely eliminating all clinical signs of infection. The porous space maintainers, designed to encourage adhesion of the oral mucosa, have a large surface area, increasing the risk of infection [26, 27]. Systemic antibiotics have poor penetration into implants and necrotic bone [28]. Implant-associated infections are difficult to treat, and treatment often involves the removal of the hardware, surgical debridement, and utilization of a new sterilized implant [29]. Ultimately, this full segmental defect model resulted in more infectious complications than other ovine mandibular defects explored by our group [3–6]. While this study was not designed to determine the cause of these surgical site infections, it is likely that the instability of this defect resulted in increased inflammation, leading to dehiscence and translocation of oral bacteria to the surgical site and hardware. In future iterations of this model, antibiotic surgical prophylaxis will be performed similar to human protocols [30], although this may not prevent subsequent infection that develops at later time points. The utilization of local antibiotic release, such as incorporation of poly(lactic-co-glycolic acid) microparticles, that are capable of extended antibiotic delivery, within the space maintainers [31], may have assisted in the treatment or prevention of such an infection. In a previous study comparing space maintainers with or without antibiotics in an ovine model, a partial segmental defect inoculated with a bioluminescent strain of *Staphylococcus aureus* and filled with a vancomycin-loaded space maintainer did not develop clinical or histological signs of a mandibular infection, while the sheep that received an unloaded space maintainer developed large dehiscence and inoculated bacteria was recovered from within the oral cavity [10]. The use of antibiotic-loaded porous space maintainers should be encouraged when available for mandibular space maintenance.

Radiographically, there were no differences between the bony tissue generated in large and small bioreactors in terms of BV/TV, TbN, TbTh, or TbSp. The BV/TV measured from the 50:50 bioreactors fell between those filled with either 100% autograft or 100% xenograft tested in a previous study [10]. Mechanical testing also demonstrated no differences in the compressive properties between the tissue formed in large and small bioreactors at 9 weeks; however, the compressive strength and modulus had increased significantly from the 4-week time point. The compressive modulus and strength were similar to previous autograft- and synthetic graft-filled bioreactors that were successfully utilized to reconstruct hemisegmental defects [3] and to harvested autograft- and xenograft-filled bioreactors [10]. The BV/TV of the tissue was significantly higher at 9 weeks (at harvest) and at 21 weeks (after transfer and time for further maturation and modeling) than it was prior to implantation, but it never reached that of the contralateral control. However, infection impacts bone growth and remodeling [32], which was observed at various time points in 4 of the 5 animals. Although vascularized bone flaps have been utilized clinically to treat osteomyelitis [33], only one of the 3 sheep that completed our study demonstrated union with the adjacent callus in multiple microCT sections. Previous sheep studies utilizing bioreactors packed with 100% xenograft demonstrated that the majority of the bone within the chambers after 9 weeks was still largely xenograft with minimal newly formed bone within the bioreactor [10]. In this study, by 21 weeks, the majority of the mineralized tissue transferred to the reconstructed mandible was newly formed bone. Other researchers have demonstrated little bone formation around Bio-Oss^®^ in short-term studies [34], while signs of degradation and mature bone surrounding the xenograft granules can be seen in studies exceeding 6 months [35–37].

In this study, a mixture of autograft and xenograft was utilized within the bioreactors. Previously, it has been shown that autograft alone formed mature bone, but xenograft alone better approximated the dimensions of the 3D-printed bioreactor space yet grew less mature bone [10]. This corroborates previous work showing lower graft coverages within the xenograft than the autograft. Tissue maturity of the bony tissue within the bioreactor was a mix of woven and cortical bone, consistent with what was seen previously [3, 10]. The larger bioreactors within this study had an additional protrusion to create a groove within the generated bone that could be used as a canal for a neurovascular bundle. The addition of this protrusion did not impact the fractional depth, indicating that the generated tissues were a good approximation of the more complex structure. A mix of autograft and xenograft allowed for the generation of a bony tissue with the desired dimensions without sacrificing maturity.

Despite large dehiscence and infection being present in several animals, the transferred tissue still has visible osteoblasts. Vascularized tissue flaps are commonly utilized in infected defects as part of a salvage treatment or prophylactically in patients who are at risk [38]. The flaps utilized in these cases tend to be myocutaneous or fasciocutaneous, which provide an ample blood supply to the affected area [39]. Despite transferring our bioreactor tissue as a vascularized flap with intercostal artery and vein, the animals still demonstrated clinical signs of infection, and histological analysis showed significant inflammatory infiltrates. Biofilms can form on segments of necrotic bone, in addition to foreign bodies (such as space maintainers, plates, or screws) when the bacteria begin producing polysaccharides and proteins which encase the bacterial cells [40]. These biofilms prevent phagocytosis by immune cells as well as confer antibiotic resistance, and its dispersal can result in re-seeding of the defect, even after hardware removal [40]. After extensive debridement, exchange of hardware, and suturing closed the mucosal dehiscence, the new sterile hardware provided a new nidus for the existing infection.

## Conclusions

In this study, a two-stage approach to craniofacial reconstruction was tested in an ovine model of a full segmental mandibular bone defect. The load-bearing defect, located immediately adjacent to the oral mucosa, was a challenging and clinically relevant defect. Hardware failure and infection were significant issues during the study; it was determined that a single lateral plate did not provide adequate fixation and that a two-plate strategy, providing both inferior and lateral support, was necessary. The large, complex bioreactors with central protuberance, as well as the smaller bioreactors, grew robust bony tissue that was easily transferred to the mandible. Several of the animals developed mandibular infection and mucosal dehiscences, creating a challenging environment for reconstruction, and variability was introduced to determine an adequate defect fixation strategy. Nevertheless, we demonstrated that the tissues transferred as a vascularized flap continued to undergo remodeling within the mandibular site, suggesting this could be a valuable strategy when combined with an antibiotic-releasing space maintainer and a fixation strategy that accounts for the motion of the mandible of the animal model.

## Supporting information

S1 Table The plate and screw arrangements for each of the 5 sheep in the study. (PNG)

S2 Table Bioreactor scoring matrices. (PNG)

S3 Table Mandible scoring matrices. (PNG)

S4 Table Radiographic MicroCT and mechanical data from sheep specimen samples taken from sheep removed from the study at unanticipated time intervals due to infection. (PNG)

S1 Fig Bioreactor designs. (A) Initial computer assisted design models with internal dimensions given in centimeters and (B) final bioreactors printed with polymethylmethacrylate filament and heat-molded with ethylene-vinyl acetate cuffs. Both bioreactors were 2 cm in length, but the larger bioreactors (left) had a protrusion to create a location within the tissue for the inferior alveolar nerve.
(PNG)

S2 Fig Schematic of sheep mandible, with area of surgery and defect creation indicated within the green box. The inferior alveolar nerve can be seen exiting through the mental foramen, and branches of the facial nerve can be seen running close to the defect site.
(PNG)

S3 Fig Histological image and data from sheep bioreactor samples taken from sheep removed at 4 weeks and mandible specimen from sheep removed at 10 weeks due to recalcitrant infections. Scale bar = 2 mm. Direction of tissue growth indicated by arrow, as bony tissue can be seen nearest the periosteal surface.
(PNG)

S4 Fig Histological images of the contralateral controls. A low magnification image (A) and higher magnification images of the intact oral mucosa (B) and new bone growth on the inferior border (C). Scale bar: A = 5 mm, B = 1 mm, C = 200 μm.
(PNG)
