# Supplementary figures and images for: Repair of complex ovine segmental mandibulectomy utilizing customized tissue engineered bony flaps
Source: PLoS One. 2023 Feb 24;18(2):e0280481. doi: 10.1371/journal.pone.0280481 (PMC9955661; doi:10.1371/journal.pone.0280481)

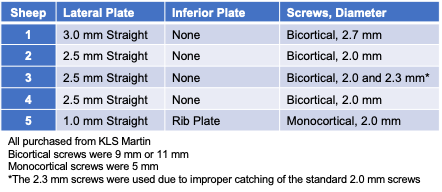

Supplement: S1 Table — (PNG) [file pone.0280481.s001.png]

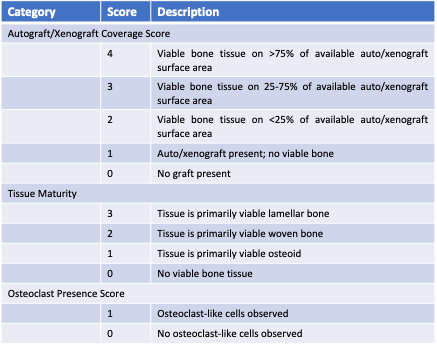

Supplement: S2 Table — (PNG) [file pone.0280481.s002.png]

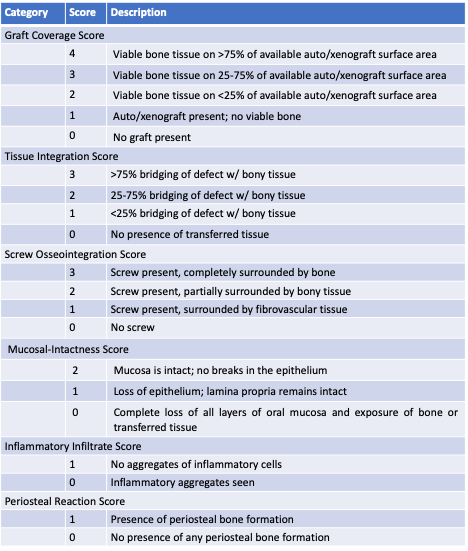

Supplement: S3 Table — (PNG) [file pone.0280481.s003.png]

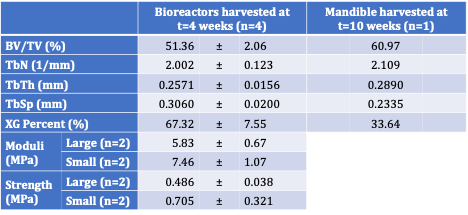

Supplement: S4 Table — (PNG) [file pone.0280481.s004.png]

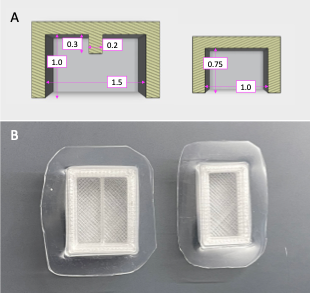

Supplement: S1 Fig — (A) Initial computer assisted design models with internal dimensions given in centimeters and (B) final bioreactors printed with polymethylmethacrylate filament and heat-molded with ethylene-vinyl acetate cuffs. Both bioreactors were 2 cm in length, but the larger bioreactors (left) had a protrusion to create a location within the tissue for the inferior alveolar nerve. (PNG) [file pone.0280481.s005.png]

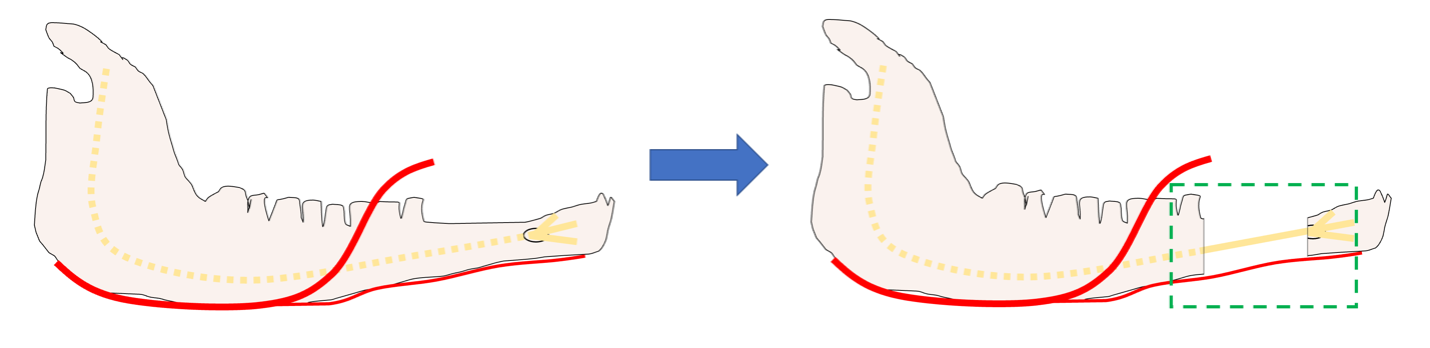

Supplement: S2 Fig — The inferior alveolar nerve can be seen exiting through the mental foramen, and branches of the facial nerve can be seen running close to the defect site. (PNG) [file pone.0280481.s006.png]

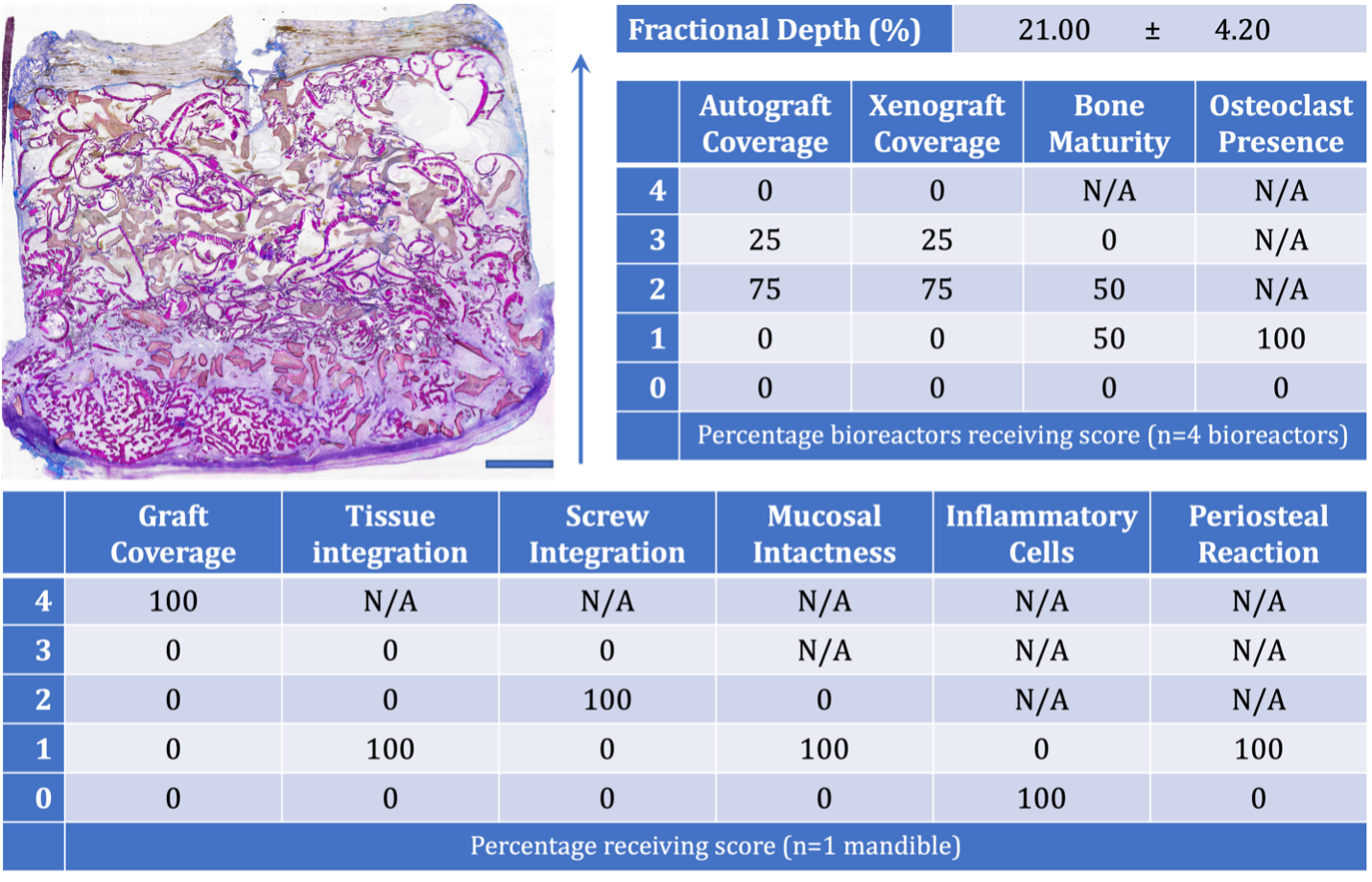

Supplement: S3 Fig — Scale bar = 2 mm. Direction of tissue growth indicated by arrow, as bony tissue can be seen nearest the periosteal surface. (PNG) [file pone.0280481.s007.png]

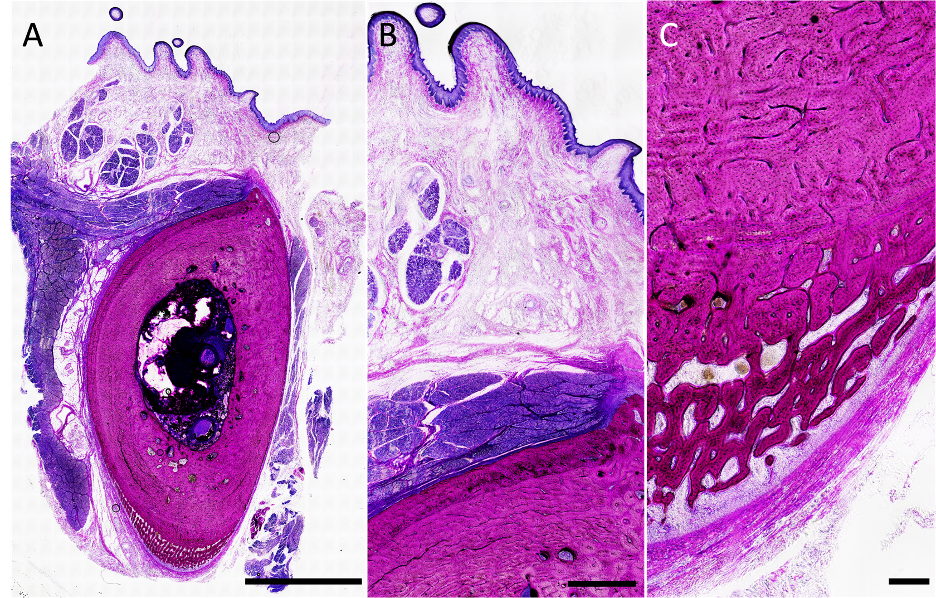

Supplement: S4 Fig — A low magnification image (A) and higher magnification images of the intact oral mucosa (B) and new bone growth on the inferior border (C). Scale bar: A = 5 mm, B = 1 mm, C = 200 μm. (PNG) [file pone.0280481.s008.png]
